# To sir with respects and affection

**Published:** 2008

**Authors:** K. Mathangi Ramakrishnan

**Affiliations:** Retired Prof and Head of Plastic Surgery, Kilpauk Medical College, Chennai, India. Email: kmr_mathangi@hotmail.com

Prof. C. R.Sundararajan, a legendary figure in the field of Plastic Surgery, was our teacher, friend and guide for over four decades. A simple man with rough exterior had a heart of gold and poured affection at every stage of our career. Indeed a teacher who always wanted to be perfect; a surgeon who would not deviate from the rules of Plastic Surgery, a true Plastic Surgeon who planned his operations well, and executed the correct procedures to suit the defect and a surgeon who did his post operative dressings himself to make his students feel that the outcome of surgery does not depend on the operative procedure alone, but also on the dressings. This was the kind of Plastic Surgeon that he was and all of us admired him.

**Figure:**
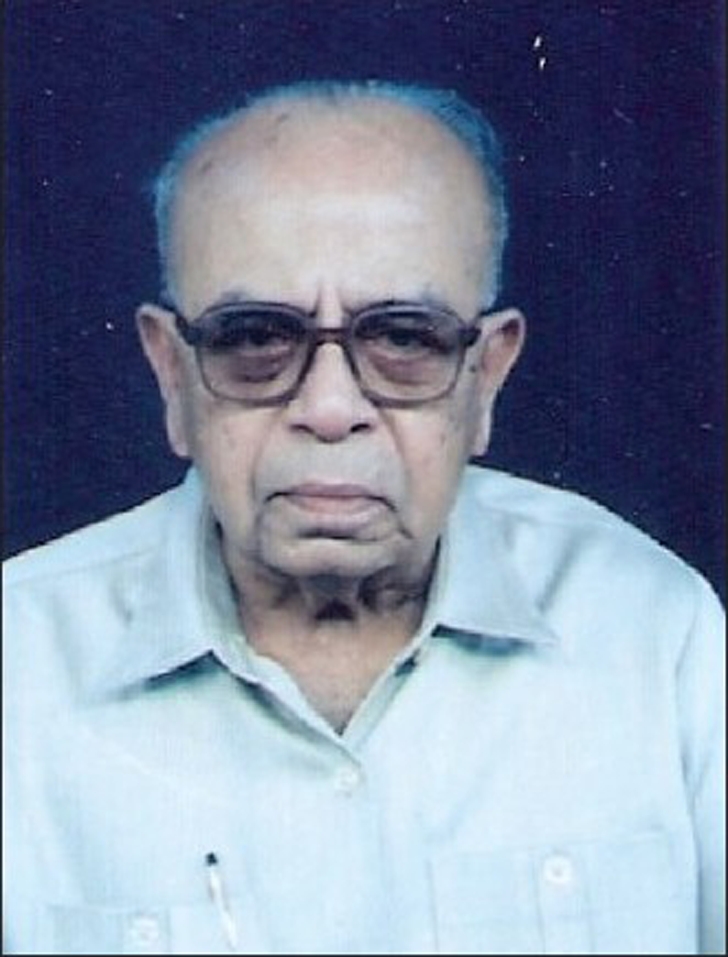
C. R. Sundararajan (1926 - 2008)

He was affectionate; never said “NO” to any academic activity; moved with all of us - young and old and during the recent visit to Varanasi for the APSICON 2008, told me that he enjoyed the meeting thoroughly.

BURNS - not altogether an attractive speciality, was one of his pet projects. I personally owe a lot for his encouragement in this field.

To feel that “SIR” is not there to talk to or see at; is a BIG VOID. Wherever he is, I am sure his guiding spirit will keep a helping hand on all the Plastic Surgeons, who went through his guidance.

We all miss him so much. “Oh GOD! Keep him in your FEET well and active for ever”.

